# A Translation System Reconstituted with Human Factors Proves That Processing of Encephalomyocarditis Virus Proteins 2A and 2B Occurs in the Elongation Phase of Translation without Eukaryotic Release Factors*

**DOI:** 10.1074/jbc.M114.593343

**Published:** 2014-09-25

**Authors:** Kodai Machida, Satoshi Mikami, Mamiko Masutani, Kurumi Mishima, Tominari Kobayashi, Hiroaki Imataka

**Affiliations:** From the ‡ Department of Materials Science and Chemistry and; § Molecular Nanotechnology Research Center, Graduate School of Engineering, University of Hyogo, Himeji 671-2201, Japan and; ¶ RIKEN Systems and Structural Biology Center, Yokohama 230-0045, Japan

**Keywords:** Protein Expression, Protein Processing, Protein Synthesis, Translation Regulation, Viral Protein, Cell-free System, Reconstitution System

## Abstract

The genomic RNA of encephalomyocarditis virus (EMCV) encodes a single polyprotein, and the primary scission of the polyprotein occurs between nonstructural proteins 2A and 2B by an unknown mechanism. To gain insight into the mechanism of 2A-2B processing, we first translated the 2A-2B region *in vitro* with eukaryotic and prokaryotic translation systems. The 2A-2B processing occurred only in the eukaryotic systems, not in the prokaryotic systems, and the unprocessed 2A-2B protein synthesized by a prokaryotic system remained uncleaved when incubated with a eukaryotic cell extract. These results suggest that 2A-2B processing is a eukaryote-specific, co-translational event. To define the translation factors required for 2A-2B processing, we constituted a protein synthesis system with eukaryotic elongation factors 1 and 2, eukaryotic release factors 1 and 3 (eRF1 and eRF3), aminoacyl-tRNA synthetases, tRNAs, ribosome subunits, and a plasmid template that included the hepatitis C virus internal ribosome entry site. We successfully reproduced 2A-2B processing in the reconstituted system even without eRFs. Our results indicate that this unusual event occurs in the elongation phase of translation.

## Teaser

**Background:** The mechanism by which the encephalomyocarditis virus protein 2A-2B is processed into 2A and 2B is unknown.

**Results:** A translation system reconstituted with defined human factors recapitulates 2A-2B processing.

**Conclusion:** 2A-2B processing occurs at the elongation step.

**Significance:** The translation system reconstituted with human factors should be a useful tool for studying eukaryotic protein synthesis mechanisms.

## Introduction

Protein translation can be dissected into four stages: initiation, elongation, termination, and ribosome recycling. The molecular mechanisms of each stage have been studied in detail using *in vitro* translation systems derived from cell extracts or reconstituted with purified factors. For prokaryotes, the protein synthesis using recombinant elements (PURE)^3^ system (1), a protein synthesis system consisting of translation factors, aminoacyl-tRNA synthetases, tRNAs, and ribosomes from *Escherichia coli*, has been successfully utilized to solve important questions regarding bacterial translation (2–4) and to create novel synthetic systems by combining with a ribozyme technology (5) or a ribosome display system (6).

Establishing a complete reconstitution system for eukaryotic translation has lagged behind prokaryote translation reconstitution partly because the initiation phase for eukaryotic translation is far more complicated than that of prokaryotes. In 1996, Pestova *et al.* (7) successfully reconstituted eukaryotic translation initiation. That system depended on the internal ribosome entry site (IRES) of encephalomyocarditis virus (EMCV), a cardiovirus of the picornavirus family, and it included purified eukaryotic translation initiation factors (eIFs) and the 40 S ribosomal subunit (7). They subsequently reconstituted the initiation phase driven by the hepatitis C virus (HCV) IRES (8) and the cap structure (9). Following successful reconstitution of these initiation events, the same group reconstituted the next steps of translation: peptide elongation and termination of eukaryotic translation (10). Finally, they established a complete system that reproduced translation initiation, peptide elongation, termination, and ribosome recycling (11, 12); however, to date, translation of a long peptide has not been attempted. Sarnow and co-workers (13) reconstituted HCV IRES-dependent translation with translation elongation factors, aminoacyl-tRNAs, and ribosomal subunits (40 and 60 S) in the presence of a relatively high concentration of magnesium to synthesize radiolabeled peptides (3–7 kDa). In that system, translation proceeded without eIFs because the 80 S ribosome could initiate translation by directly binding to the HCV IRES under the conditions used (13).

The genome of EMCV is a single-stranded positive-sense RNA of 7.9 kb. Upon infection, the EMCV RNA is translated into a single polyprotein, which is subsequently processed into structural (capsid) and nonstructural proteins mostly through the actions of the virally encoded protease 3C (14). The primary event in processing the EMCV polyprotein is the cleavage that occurs between nonstructural proteins 2A (144 amino acids) and 2B (148 amino acids) independently of the 3C protease (15). Studies of foot-and-mouth disease virus (FMDV), an aphthovirus of the picornavirus family, demonstrated that the C-terminal 18 amino acids of 2A plus the first amino acid (proline) of 2B are responsible for this processing event (16, 17). Site-directed mutagenesis of these 19 amino acids in EMCV and FMDV revealed that the amino acid sequence, NPGP, is critically important for this processing event; NPG are the C-terminal three amino acids of 2A, and the last P represents the first amino acid of 2B (15–17). Based on results from experiments with *in vitro* translation systems (a rabbit reticulocyte lysate and a wheat germ extract), Donnelly *et al.* (18) proposed a model in which the C-terminal 18-amino acid sequence of 2A modulates the activity of the peptidyltransferase center (PTC) of the ribosome. Thus, the peptidyl (2A)-tRNA^Gly^ ester linkage is hydrolyzed before it forms a peptide bond with the prolyl-tRNA in the A site (18). Experiments in a yeast system substantiated that hypothesis and suggested that eukaryotic release factors 1 and 3 (eRF1 and eRF3) played a key role in the reaction (19).

The present study aimed to define the translation factors that are required for 2A-2B processing. We reconstituted the HCV IRES-dependent protein synthesis system with eukaryotic elongation factor (eEF) 1, eEF2, eRF1, eRF3, aminoacyl-tRNA synthetases, tRNAs, and ribosomes and demonstrated that 2A-2B processing did not require eRFs.

## EXPERIMENTAL PROCEDURES

### Construction of Plasmids

#### 

##### Translation Factors

Complementary DNAs (cDNAs) of the human genes eEF1A, eEF1Bα, eEF1Bγ, eRF1, and eRF3 were obtained by reverse transcription followed by PCR (RT-PCR) using human placenta RNA (Clontech). DNA primers for RT-PCR were chosen based on the reported sequences (GenBank^TM^ accession numbers NM_001402 for eEF1A, X60489 for eEF1Bα, Z11531 for eEF1Bγ, NM_004730 for eRF1, and NM_002094 for eRF3. eEF1A and eEF1Bγ cDNAs were cloned into the EMCV IRES-dependent expression vector pUC-T7-EMCV-His-MCS-ter (20) to generate pUC-T7-EMCV-His-eEF1A and pUC-T7-EMCV-His-eEF1Bγ, respectively. The eEF1Bα cDNA was cloned into pUC-T7-EMCV-MCS-ter (20) to construct pUC-T7-EMCV-eEF1Bα. eRF1 and eRF3 cDNAs were cloned into pGEX6P (GE Healthcare) to construct pGEX6P-eRF1 and pGEX6P-eRF3, respectively.

##### Aminoacyl-tRNA Synthetases (ARSs)

The cDNAs of human methionyl-tRNA synthetase (MetRS), aspartyl-tRNA synthetase (AspRS), glutamyl-prolyl-tRNA synthetase (Glu-ProRS), alanyl-tRNA synthetase (AlaRS), cysteinyl-tRNA synthetase (CysRS), phenylalanyl-tRNA synthetase (PheRS) β subunit, valyl-tRNA synthetase (ValRS), and tyrosyl-tRNA synthetase (TyrRS) were obtained by RT-PCR using human placenta poly(A) RNA and DNA primers based on the reported sequences (GenBank accession numbers P56192 for MetRS, DQ890741 for AspRS, NM_004446 for Glu-ProRS, DQ891634 for AlaRS, DQ890773 for CysRS, NM_005687 for PheRS β subunit, NM_006295 for ValRS, and DQ891666 for TyrRS). These ARS cDNAs were inserted into the pUC-T7-EMCV-GST plasmid (20) to construct pUC-T7-EMCV-GST-MetRS, -AspRS, -Glu-ProRS, -AlaRS, -CysRS, -ValRS, and -TyrRS. The cDNAs of the human histidyl-tRNA synthetase (HisRS), tryptophanyl-tRNA synthetase (TrpRS), asparaginyl-tRNA synthetase (AsnRS), PheRS α subunit, seryl-tRNA synthetase (SerRS), glycyl-tRNA synthetase (GlyRS), glutaminyl-tRNA synthetase (GlnRS), threonyl-tRNA synthetase (ThrRS), isoleucyl-tRNA synthetase (IleRS), arginyl-tRNA synthetase (ArgRS), leucyl-tRNA synthetase (LeuRS), and lysyl-tRNA synthetase (LysRS) in the pF1KM plasmid were purchased from Promega (21). These cDNAs were digested out from each plasmid with restriction enzymes SgfI and PmeI and incorporated into the plasmid pUC-T7-EMCV-GST SgfI/PmeI, a derivative of the pUC-T7-EMCV-GST plasmid to construct pUC-T7-EMCV-GST-HisRS, -TrpRS, -AsnRS, -SerRS, -GlyRS, -GlnRS, -ThrRS, -IleRS, -ArgRS, -LeuRS, and -LysRS. The cDNAs for PheRS α and β subunits were incorporated into pUC-T7-EMCV-His-MCS-ter (20) to generate pUC-T7-EMCV-His-PheRSα and pUC-T7-EMCV-His-PheRSβ, respectively.

##### Template Plasmids

A hemagglutinin (HA) sequence was inserted into the HCV IRES-dependent expression vector pUC-T7-HCV-MCS-ter (20) to generate pUC-T7-HCV-HA. A DNA fragment encoding the EMCV 2A-2B region (2A, 144 amino acids; 2B, 148 amino acids) with a FLAG or HA tag at the C terminus was amplified by PCR using the plasmid pUC18 EMCV Rbz (22) as a template. After digestion with BamHI and XhoI, the DNA fragment was incorporated into the plasmid pUC-T7-HCV-HA to generate pUC-T7-HCV HA-2A-2B-FLAG or pUC-T7-HCV HA-2A-2B-HA. To express the 2A-2B region with the reticulocyte lysates, wheat germ extracts, and bacterial S30 and PURE systems, the HA-2A-2B-FLAG DNA was inserted into the corresponding vectors that were provided in protein expression kits. The coding regions of *Renilla* luciferase (Rluc), β-galactosidase (β-gal) (20), poly(A)-binding protein (PABP) (23), and β-actin (24) were inserted into the plasmid pUC-T7-HCV-HA to generate the templates for expression of each protein with the HA tag at the N terminus. To construct plasmids for expression of C-terminally HA-tagged proteins, the coding regions of Rluc, β-actin, PABP, and β-gal with the HA tag at the C terminus were inserted into the plasmid pUC-T7-HCV-MCS-ter (20).

##### N-terminal Deletions of 2A

DNA fragments spanning the C-terminal 14, 15, 16, 17, 18, 19, and 20 amino acids of 2A to 2B-FLAG were amplified by PCR and incorporated into the plasmid pUC-T7-HCV-MCS-ter (20) to construct pUC-T7-HCV 2A (14, 15, 16, 17, 18, 19, and 20 aa)-2B-FLAG, respectively.

##### Alanine Substitutions

Overlap PCR was used to change each amino acid of the C-terminal 18 amino acids of 2A and the N-terminal four amino acids of 2B into alanine in the plasmid pUC-T7-HCV 2A (18 aa)-2B-FLAG.

##### Silent Mutations

The wild type nucleotide sequence (aat cca ggg ccc for NPGP) was mutated by overlap PCR into silent-1 (aac ccc gga cca) and silent-2 (aac cct ggt cct) sequences in the plasmid pUC-T7-HCV HA-2A-2B-HA to generate pUC-T7-HCV HA-2A(silent-1)2B-HA and pUC-T7-HCV HA-2A(silent-2)2B-HA, respectively. Neither mutation alters the amino acid sequence.

##### Other Constructs

To construct the plasmid pUC-T7-HCV HA-2A-HA-2B (22 aa), the following mutations were introduced in pUC-T7-HCV HA-2A-2B by PCR with appropriate primers. The N-terminal amino acid sequence of 2B was changed from PFMFRPRKQVFQTQGAA to PFEYDYDVPDYAGQGAA where the HA sequence is underlined, and the C-terminal 22 amino acids of 2B were replaced with amino acids AHYAGYFADLLIHDIETNPGPF without a stop codon. To construct the plasmid pUC-T7-HCV HA-2A(uORF2) for producing a tRNA-conjugated peptide, the C-terminal 22 amino acids of 2A were replaced with amino acids MQPLVLSAKKLSSLLTCKYIPP with a termination codon (TAA).

### Purification of Proteins

#### 

##### eEF1

Three eEF1 DNA sequences (eEF1A, eEF1Bγ, and eEF1Bα) carried in expression vectors pUC-T7-EMCV-His-eEF1A, pUC-T7-EMCV-His-eEF1Bγ, and pUC-T7-EMCV-eEF1Bα were co-transfected into BHK-21 cells that were infected with a vaccinia virus (LO-T7-1), which conferred expression of T7 RNA polymerase, as described previously (24, 25). Two days later, cells were harvested and frozen at −85 °C. The frozen cells (∼2 g) were suspended in 30 ml of buffer (20 mm HEPES-KOH, pH 7.5, 100 mm KCl, 10% glycerol, 2 mm 2-mercaptoethanol, 1 mm EDTA, 0.1% Triton X-100, 10 mm imidazole, one tablet of a mixture of protease inhibitors; Nacalai). The cells were lysed by vortexing and centrifuged at 15,000 × *g* for 30 min at 4 °C. The supernatant was applied to a nickel-nitrilotriacetic acid resin (bed volume, 0.4 ml; Qiagen), and unbound proteins were removed by washing with 4 ml of buffer (20 mm HEPES-KOH, pH 7.5, 500 mm KCl, 10% glycerol, 2 mm 2-mercaptoethanol, 1 mm EDTA, 0.1% Triton X-100, 10 mm imidazole). Then the resin was washed with 4 ml of the same buffer but with the KCl concentration reduced to 100 mm. The eEF1 complex, which consisted of His-eEF1A, His-eEF1Bγ, and eEF1Bα, was eluted in a stepwise manner with increasing concentrations of imidazole (50, 100, 250, and 1000 mm) in 1.2 ml of buffer (0.1 m KCl, 20 mm HEPES-KOH, pH 7.5, 10% glycerol). The eluates with 50, 100, and 250 mm imidazole were combined and loaded onto a HiPrep 16/60 Sephacryl S-300 HR gel filtration column (120 ml; GE Healthcare) equilibrated with buffer (20 mm HEPES-KOH, pH 7.5, 100 mm KCl, 10% glycerol). Elution was carried out with the same buffer at a flow rate of 0.5 ml/min, and 1.5-ml fractions were collected with the ÄKTAprime plus system (GE Healthcare). The fractions that contained the eEF1 complex were combined and concentrated with Amicon Ultra-15 (molecular weight cutoff, 30,000; Millipore) to ∼0.1 ml. The final protein concentration of the sample was 15–30 mg/ml.

##### eEF2

HeLa S3 cells (3–4 × 10^9^) were suspended in buffer (10 ml; 20 mm HEPES-KOH, pH 7.5, 50 mm KCl, 50 mm potassium acetate, 2 mm magnesium acetate, 2 mm dithiothreitol) and disrupted by nitrogen pressure (1 megapascal for 30 min) in a Mini-Bomb cell disruption chamber (Kontes) on ice. The lysate was centrifuged at 15,000 × *g* for 20 min at 4 °C, and the supernatant was again centrifuged at 15,000 × *g* for 20 min at 4 °C. This supernatant was centrifuged at 25,000 rpm in an SW41 rotor (Beckman Coulter) for 16 h at 4 °C. The supernatant was used for tRNA purification (see “Purification of tRNAs”). The pellet (crude ribosomal pellet) was suspended in buffer (8 ml; 20 mm HEPES-KOH, pH 7.5, 50 mm KCl, 5 mm magnesium acetate, 0.25 m sucrose) by mixing with a magnetic stirrer for 4 h on ice. After addition of 3 m KCl (1.6 ml), stirring was further continued for 2 h, and the sample was then centrifuged at 25,000 rpm in an SW41 rotor for 16 h at 4 °C. The supernatant was mixed with ammonium sulfate to 50% saturation and centrifuged. The supernatant was brought to 70% saturation in ammonium sulfate, and precipitates were collected by centrifugation. The pellet was dissolved in buffer (2.5 ml; 20 mm HEPES-KOH, pH 7.5, 50 mm KCl, 1 mm DTT, 1 mm EDTA, 10% glycerol) and passed through a PD-10 column equilibrated with the same buffer. The eluate (3.5 ml) was loaded onto a Q-Sepharose resin (0.3 ml; GE Healthcare) equilibrated with the same buffer. After being washed with the same buffer (3 ml), proteins were eluted out in a stepwise manner that increased the concentration of KCl from 100 to 1000 mm in buffer (0.9 ml each; 20 mm HEPES-KOH, pH 7.5, 1 mm DTT, 1 mm EDTA, 10% glycerol). Eluates with 100 and 200 mm KCl were combined and passed through a PD-10 column equilibrated with the same buffer containing 50 mm KCl. The eluate was then applied to a phosphocellulose column (P11; 0.2 ml) equilibrated with the same buffer containing 50 mm KCl. After washing with the same buffer (2 ml), proteins were eluted out with buffer (0.6 ml; 20 mm HEPES-KOH, pH 7.5, 1 mm DTT, 1 mm EDTA, 10% glycerol) with an increasing concentration of KCl (100, 200, 300, 500, and 1000 mm). Eluates with 100 and 200 mm KCl were combined and concentrated with Amicon Ultra-15 (molecular weight cutoff, 50,000) to ∼0.05–0.1 ml. The final concentration of the sample was 2.0–3.5 mg/ml. The presence of eEF2 in each fraction was monitored by Western blotting with anti-eEF2 antibody (Cell Signaling Technology).

##### eRF1 and eRF3

GST-eRF1 and GST-eRF3 were expressed in bacterial strain BL-21(DE3) transformed with pGEX6P-eRF1 and pGEX6P-eRF3, respectively, and purified on glutathione-Sepharose 4B resin (GE Healthcare) as described (26). Non-tagged eRF1 and eRF3 proteins were cleaved from the GST tag with PreScission protease (GE Healthcare) as described (26). Chromatography with SP-Sepharose (GE Healthcare) was used to complete purification of eRF3. The final concentration of each sample was 0.8–1.0 mg/ml.

##### Aminoacyl-tRNA Synthetase

Because eight ARSs (Glu-ProRS, IleRS, LeuRS, MetRS, GlnRS, LysRS, ArgRS, and AspRS) exist as a multiprotein complex (27), we expressed these ARSs (GST-tagged form) simultaneously in LO-T7–1-infected BHK-21 cells by co-transfecting the plasmids pUC-T7-EMCV-GST-Glu-ProRS, -IleRS, -LeuRS, -MetRS, -GlnRS, -LysRS, -ArgRS, and -AspRS as described previously (24). The transfected BHK-21 cells were lysed, and the extract was passed through glutathione-Sepharose 4B resin. Then a mixture of these ARSs was obtained by removing the GST tags with PreScission protease as described previously (28). We did not co-transfect the associated proteins (p43, p38, and p18) (27) because in preliminary experiments co-expression of p43, p38, and p18 with the ARSs did not enhance the yield or activity of recombinant ARSs. Ten of the remaining ARSs were individually expressed in the LO-T7–1-infected BHK-21 cells by transfection with pUC-T7-EMCV-GST-AlaRS, -CysRS, -GlyRS, -HisRS, -AsnRS, -SerRS, -ThrRS, -ValRS, -TrpRS, or -TyrRS. The GST tags were removed from these ARSs individually as described above. PheRSα and PheRSβ were expressed with His tags in the vectors pUC-T7-EMCV-His-PheRSα and pUC-T7-EMCV-His-PheRSβ. These constructs were co-transfected into LO-T7–1-infected BHK-21 cells, and a mixture of His-PheRSα and His-PheRSβ was purified as described for eEF1. T7 RNA polymerase was expressed in *E. coli* and purified as described previously (20).

### Purification of tRNAs

After centrifugation of the disrupted HeLa S3 cells (see “eEF2”), the supernatant was mixed with an equal volume of Sepazol-RNA I (Nacalai) and kept at room temperature for 10 min. After addition of 15 volume (relative to that of Sepazol-RNA I) of chloroform, the mixture was centrifuged at 15,000 × *g* for 15 min at 4 °C. The upper layer was mixed with an equal volume of isopropanol and kept at room temperature for 5 min. The mixture was centrifuged at 15,000 × *g* for 10 min at 4 °C, and the pellet was dissolved in water (10 ml). After addition of 3 m sodium acetate (1.1 ml; pH 7.0) and isopropanol (6 ml), the sample was centrifuged at 15,000 × *g* for 15 min at 4 °C. The supernatant (17 ml) was mixed with isopropanol (4.9 ml) and kept at room temperature for 20 min. The mixture was centrifuged at 15,000 × *g* for 20 min at 4 °C, and the pellet was kept at −80 °C as the crude tRNA fraction until use. Pure tRNAs were purified from the crude tRNA fraction as follows. The crude tRNA (1–2 mg) was dissolved in Gel Loading Buffer II (0.2 ml; Ambion) and resolved on a 12% polyacrylamide, 8 m urea gel. The area of the gel that was supposed to contain tRNA, as judged by the size (around 80 bases), was excised and minced into small pieces. Extraction of tRNA from the gel pieces was carried out using buffer (3 ml; 2 m ammonium acetate, 1% (w/v) SDS) twice followed by precipitation with ethanol.

### Purification of Ribosomes

The crude ribosomal pellet (see “eEF2”) was suspended in buffer (8 ml; 20 mm Tris-HCl, pH 7.5, 100 mm KCl, 4 mm magnesium acetate, 2 mm DTT) by mixing with a magnetic stirrer for 3 h on ice. After stirring, the suspension was centrifuged at 15,000 × *g* for 10 min at 4 °C, and the supernatant was again centrifuged at 15,000 × *g* for 15 min at 4 °C. The supernatant was mixed with 120 volume of 20 mm puromycin and incubated for 10 min on ice and then for 10 min at 37 °C. The incubated mixture was supplemented with 16 volume of buffer (20 mm Tris-HCl, pH 7.5, 3 m KCl, 4 mm magnesium acetate, 2 mm DTT, 0.1 mm puromycin, 10% (w/v) sucrose) and resolved by 15–30% (w/v) sucrose gradient centrifugation with buffer (20 mm Tris-HCl, pH 7.5, 0.5 m KCl, 4 mm magnesium acetate, 2 mm DTT, 0.1 mm puromycin) at 25,000 rpm in an SW28 rotor for 16 h at 4 °C. Two-milliliter fractions (fractions 1–18 or 19) were successively taken from the top of the gradient, and an aliquot of each fraction was resolved on a formaldehyde-agarose gel and stained with ethidium bromide to monitor the 18 and 28 S ribosomal RNAs, which represent the presence of 40 and 60 S subunits, respectively. The fractions that mainly contained the 40 S subunit were combined and concentrated using Amicon Ultra-15 (molecular weight cutoff, 50,000) to ∼1 ml. The fractions that mainly contained the 60 S subunits were also concentrated in the same manner. These 40 and 60 S subunit samples were separately resolved by 15–30% (w/v) sucrose gradient centrifugation in the same conditions as described above but without puromycin. The fractions that contained the 40 S subunit only and the 60 S subunit only were separately dialyzed against a 100× volume of buffer (20 mm Tris-HCl, pH 7.5, 100 mm KCl, 2 mm magnesium acetate, 2 mm DTT) for 5 h at 4 °C and then against a new batch of the same buffer overnight. The samples were concentrated as stated above. The final concentrations of the 40 and 60 S subunits were 80 and 160 absorbance (*A*) units at 260 nm, respectively.

### Reconstitution System

To reconstitute translation, the following components were mixed in a test tube: 0.6 μl of tRNAs (16–18 mg/ml), 1.7 μl of eEF1 (15–30 mg/ml), 0.3 μl of eEF2 (2.0–3.5 mg/ml), 0.5 μl of eRF1 and eRF3 (0.5 mg/ml each), 1 μl of 40 S ribosomal subunit (80 *A* units), 1 μl of 60 S ribosomal subunit (160 *A* units), 0.5 μl of T7 RNA polymerase (1 mg/ml) (20), and 0.5 μl of a human ARS mixture of 11 ARSs (AlaRS, CysRS, PheRS (α + β), GlyRS, HisRS, AsnRS, SerRS, ThrRS, ValRS, TrpRS, and TyrRS at 0.12 mg/ml each). The reaction also included a mixture of eight ARSs (0.88 mg/ml; Glu-ProRS, IleRS, LeuRS, MetRS, GlnRS, ArgRS, LysRS, and AspRS) and 1 μl of 20 amino acids (1 mm each). Additionally, we added 1 μl of 10× transcription/translation buffer (0.5 m HEPES-KOH, pH 7.5, 0.55 m potassium acetate, 60 mm magnesium acetate, 2 mm spermidine, 0.6 mg/ml creatine kinase, 200 mm creatine phosphate, 10 mm DTT, 12.5 mm ATP, 8.3 mm each of GTP, UTP, and CTP) and 1.4 μl of water. Finally, we added 0.5 μl of a template plasmid (0.5 mg/ml), and the mixture (10 μl) was incubated for up to 3 h at 32 °C.

### Other Cell-free Translation Systems

We used the ThermoScientific 1-Step Human *In vitro* Protein Expression kit (ThermoScientific Pierce), which is derived from a HeLa cell extract, the TnT Coupled Reticulocyte Lysate System (Promega), the TnT Coupled Wheat Germ Extract System (Promega), the *E. coli* T7 S30 Extract System (Promega), and the PURE system (New England Biolabs). All assays were performed according to the manufacturers' instructions. For a human cell extract-derived protein expression system, we also used a homemade HeLa cell extract-dependent system (20, 29). The results were essentially the same as those obtained with the commercial kit. In all of these systems, the total volume for one reaction was set to 18 μl, the solution contained 0.2–0.3 μg of template plasmid, and the incubation was carried out for 2 h at 25–37 °C depending on the system. In some experiments, a HeLa cell extract (1 μl; 0–40 μg of protein) was added to the PURE system reaction (18 μl) before or after translation at 37 °C.

### Analysis of Products

Samples were resolved by SDS-PAGE and analyzed by Western blotting with anti-HA (Abcam) and anti-FLAG (Sigma) antibodies based on standard techniques. Protein bands were detected and analyzed using the ImageQuant LAS 4000 mini with MultiGauge software version 3.0 (Fujifilm). When the pUC-T7-HCV-HA-Rluc-FLAG vector was used as template, 2 μl from the sample (10 μl) was subjected to an Rluc assay from the Dual-Luciferase Reporter Assay System (Promega). In some experiments (see Fig. 6, *B* and *D*, *right panel*), samples were passed through nickel-nitrilotriacetic acid resin before SDS-PAGE to remove a large amount of the eEF1 complex because this complex could disturb the resolution of products on Western blots.

## RESULTS

### 

#### 

##### 2A-2B Processing Is Eukaryote-specific and Co-translational

**FIGURE 1.**
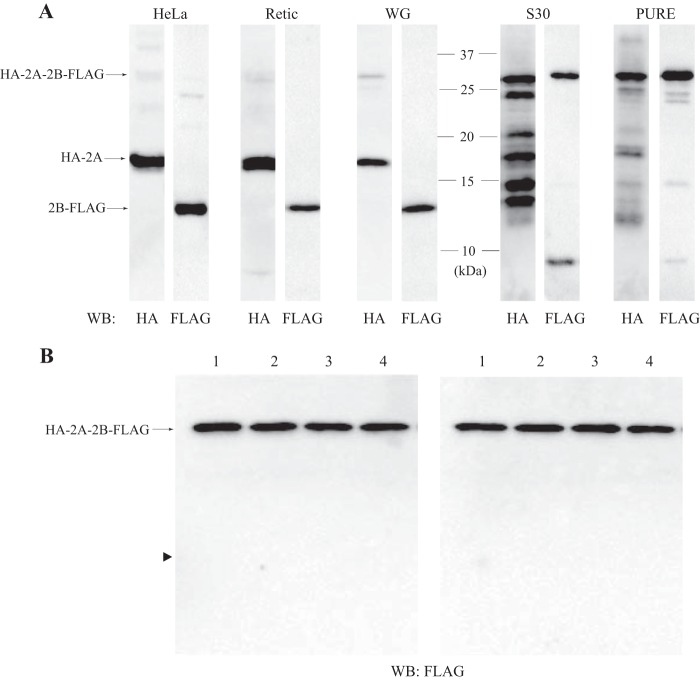
**Processing of 2A-2B is a eukaryote-specific, co-translational event.** *A*, Western blots (*WB*) probed with anti-HA or anti-FLAG antibody show protein products from HA-2A-2B-FLAG plasmids translated in eukaryotic (*HeLa*, HeLa cell extract; *Retic*, rabbit reticulocyte lysate; *WG*, wheat germ extract) and prokaryotic (*S30*, bacterial extract; *PURE*, PURE system) transcription/translation systems. *B*, Western blots probed with the anti-FLAG antibody show protein products after the PURE system (18 μl) was incubated with the plasmid encoding HA-2A-2B-FLAG in the presence of a HeLa cell extract (1 μl; *lanes 1*, buffer alone; *lanes 2*, 10 μg of protein; *lanes 3*, 20 μg of protein; *lanes 4*, 40 μg of protein). The HeLa cell extract was added to the system before (*left panel*) or after (*right panel*) the translation reaction. In the latter case, incubation was further continued for 2 h at 37 °C. The *arrowhead* indicates the position of 2B-FLAG.

To examine the mechanism of EMCV 2A-2B processing, various cell-free transcription/translation systems derived from both eukaryotic and prokaryotic cells were programmed with plasmids that encoded the 2A-2B sequence with an N-terminal HA tag and a C-terminal FLAG tag (HA-2A-2B-FLAG). The translated protein products were resolved by SDS-PAGE and analyzed by Western blotting with anti-HA and anti-FLAG antibodies. The HA-2A and 2B-FLAG proteins were separately generated in the eukaryotic systems derived from a HeLa cell extract, a rabbit reticulocyte lysate, and a wheat germ extract (Fig. 1*A*). In contrast, two prokaryotic cell-free translation systems, S30 (derived from bacterial lysate) and PURE (reconstituted with bacterial translation factors), failed to generate individual 2A and 2B proteins. Instead, they predominantly yielded the unprocessed product (HA-2A-2B-FLAG). Although the S30 system generated several anti-HA-reactive bands that migrated faster than HA-2A-2B-FLAG, this presumably resulted from premature translation termination (Fig. 1*A*). These results suggested that the 2A-2B processing of EMCV was a eukaryote-specific event, confirming previous results (30).

To examine whether a protease was present in the lysate of eukaryotic cells that could cleave the 2A and 2B product, we added HeLa cell extract to the PURE system before or after translation of HA-2A-2B-FLAG (The template used in this experiment was for expression in prokaryotes but not for eukaryotes. Therefore, the translational apparatus derived from HeLa extract is not able to produce any protein therein.). No processing of 2A-2B occurred in either case (Fig. 1*B*). This result suggested that 2A-2B processing was not due to proteolysis in the cytoplasm. We reasoned that it must occur during translation, and it might involve the eukaryotic ribosome.

##### Important Amino Acids in 2A-2B Processing

**FIGURE 2.**
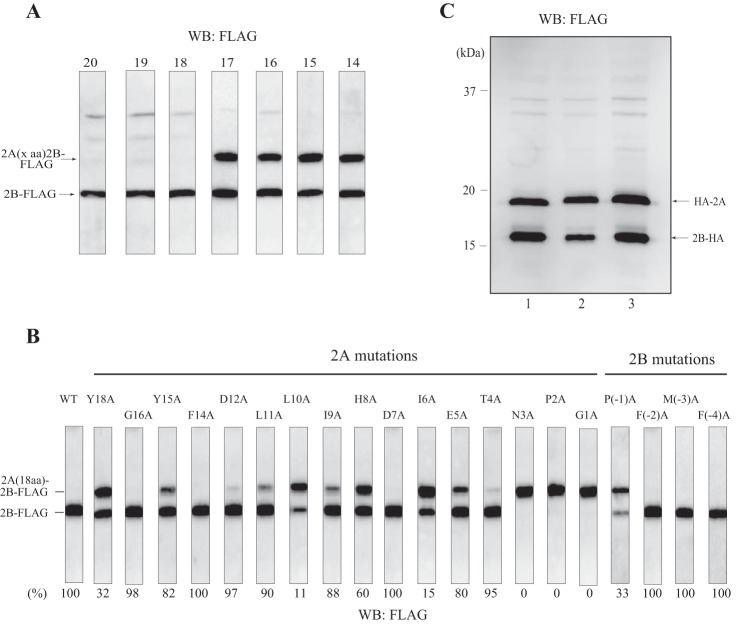
**Amino acids important for 2A-2B processing.** *A*, the HeLa cell extract-derived protein expression system was programmed with a template encoding an N-terminally truncated 2A-2B-FLAG, pUC-T7-HCV 2A (14, 15, 16, 17, 18, 19, or 20 aa)-2B-FLAG. The *number* above each panel represents the number of amino acids of 2A after truncation. Western blotting (*WB*) was performed with anti-FLAG antibody. *B*, templates bearing an alanine substitution for each of the C-terminal 18 amino acids of 2A (except two native alanine residues) and for the N-terminal four amino acids of 2B in the plasmid pUC-T7-HCV 2A (18 aa)-2B-FLAG were incubated in the HeLa cell extract-derived protein synthesis system. Western blotting was performed with anti-FLAG antibody. Each amino acid is numbered from the C-terminal amino acid (glycine) of 2A, which is set to +1. The efficiency of processing (2B-FLAG/2B-FLAG + 2A (18 aa)-2B-FLAG) is reported below each panel. *C*, plasmids pUC-T7-HCV HA-2A-2B-HA (*lane 1*), pUC-T7-HCV HA-2A(silent-1)2B-HA (*lane 2*), and pUC-T7-HCV HA-2A(silent-2)2B-HA (*lane 3*) were incubated in the HeLa cell extract-derived protein synthesis system. Western blotting was performed with anti-HA antibody.

To delineate the minimal region of 2A-2B needed for processing, we created serial truncations that removed different lengths of the N-terminal region of 2A in the HA-2A-2B-FLAG construct. Translation in the HeLa cell extract showed that the C-terminal 18 amino acids of 2A were the minimum required for processing (Fig. 2*A*). Next, we created alanine substitutions for each of these 18 amino acids (except two native alanine residues) and for the first four amino acids in 2B. The results verified the importance of the NPGP sequence (the glycine residue is the last amino acid of 2A) for processing (Fig. 2*B*) (15, 18). We then tested silent mutations for NPGP in which the nucleotide sequence was changed but the NPGP amino acids were maintained. These silent mutations showed full processing efficiency (Fig. 2*C*). Thus, it was highly unlikely that the mRNA sequence for NPGP was involved in processing.

##### 2A Is Released from tRNA

**FIGURE 3.**
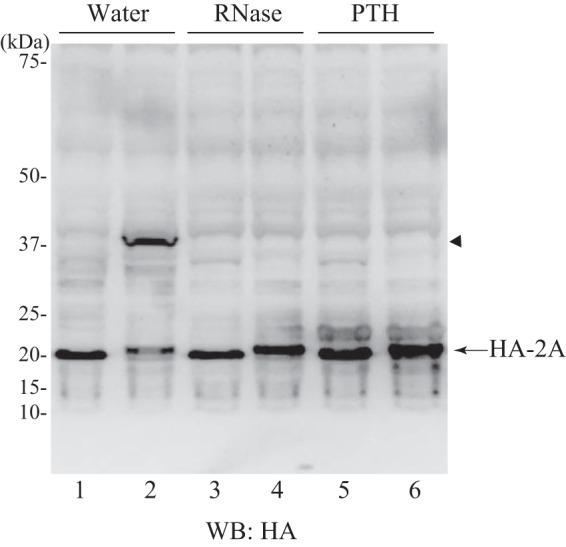
**2A is released from tRNA.** The HeLa cell extract-derived protein expression system was programmed with pUC-T7-HCV HA-2A-2B-FLAG for *lanes 1*, *3*, and *5* or with pUC-T7-HCV HA-2A(uORF2) for *lanes 2*, *4*, and *6*. After translation (10 min), samples were treated with EDTA (19.2 mm) for 5 min at 25 °C and then supplemented with magnesium acetate (22.3 mm). Each sample was subsequently treated with water (*lanes 1* and *2*), RNase A (0.1 μg/μl) (*lanes 3* and *4*), or peptidyl-tRNA hydrolase (*PTH*) (0.42 μg/μl) (*lanes 5* and *6*) for 30 min at 25 °C and resolved by NuPAGE followed by Western blotting (*WB*) with anti-HA antibody. The *arrowhead* indicates the band that disappeared due to RNase or peptidyl-tRNA hydrolase treatment.

The 2A fragment might be generated due to a translational pause of the ribosome at the C terminus of the 2A region. In that case, the 2A protein should remain ligated to the tRNA^Gly^ in the ribosomal P site as demonstrated in SecM-mediated ribosomal stall (31), translational pause in the XBP1u mRNA (32), and ribosomal arrest mediated by the upstream open reading frame 2 (uORF2) of the cytomegalovirus UL 4 gene (33). To examine this, we incubated the HeLa cell extract with the HA-2A-2B-FLAG construct and resolved the product with NuPAGE (Invitrogen; the pH of the gel is neutral) (34) to maximally retain the esterified bond between the 3′-end of the tRNA and the C terminus of the 2A peptide. Western blotting with anti-HA antibody detected the 2A peptide (Fig. 3, *lane 1*). We tested a positive control with a plasmid encoding HA-2A(uORF2) in which the C-terminal 22 amino acids of 2A had been replaced with uORF2 of the cytomegalovirus UL 4 gene (33). From this template, a product that migrated slower than the HA-2A peptide was generated (*lane 2*). Treatment with RNase A eliminated this slowly migrating product (*lane 4*). This product was also abolished by treatment with peptidyl-tRNA hydrolase (Ref. 35; kindly provided by Dr. Nagao) (we confirmed that the peptidyl-tRNA hydrolase preparation used in this experiment was devoid of an RNase activity that would decompose the body of tRNA; data not shown), indicating that the peptide was conjugated to tRNA. From the HA-2A-2B-FLAG construct, neither RNase A- nor peptidyl-tRNA hydrolase-sensitive product was produced (Fig. 3, *lanes 1*, *3*, and *5*). Thus, the 2A peptide was dissociated from tRNA despite the absence of a termination codon.

##### A Protein Expression System Reconstituted with Human Factors

**FIGURE 4.**
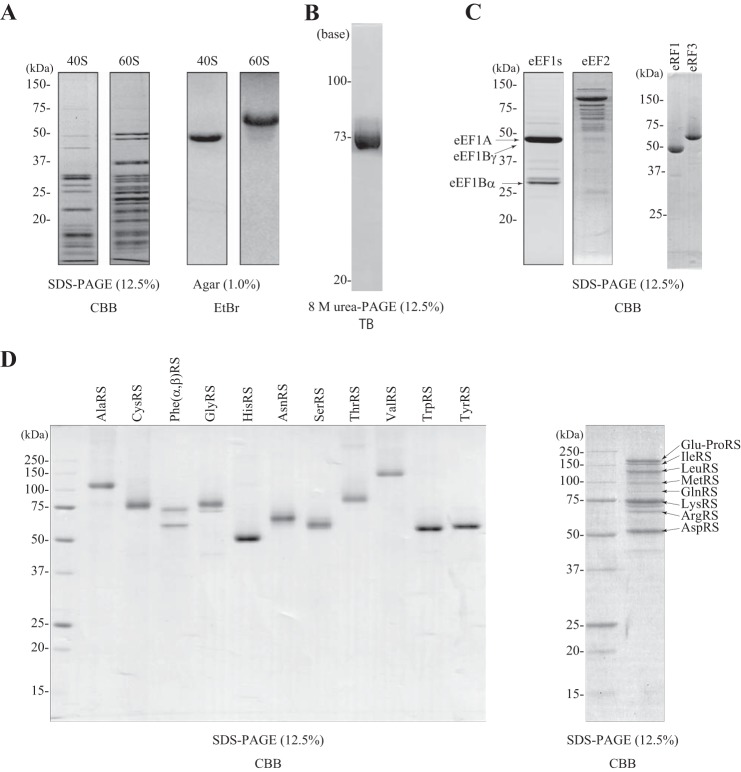
**Purification of each component used in the reconstituted protein synthesis system.** *A*, ribosomes. 40 and 60 S ribosomal subunits were resolved by SDS-PAGE (12.5%) and stained with Coomassie Brilliant Blue (*CBB*) (*left two panels*) or resolved on a denatured agarose gel (1%) and stained with ethidium bromide (*right two panels*). *B*, tRNAs. tRNAs (2 μg) were resolved by 8 m urea PAGE (12.5%) and stained with toluidine blue (*TB*). *C*, translation factors. eEF1s (eEF1A, eEF1Bα, and eEF1Bγ), eEF2, eRF1, and eRF3 (1.5 μg each) were resolved by SDS-PAGE (12.5%) and stained with Coomassie Brilliant Blue. *D*, *left panel*, AlaRS, CysRS, PheRS (α + β), GlyRS, HisRS, AsnRS, SerRS, ThrRS, ValRS, TrpRS, and TyrRS (1 μg each). *Right panel*, a mixture of Glu-ProRS, IleRS, LeuRS, MetRS, GlnRS, ArgRS, LysRS, and AspRS (8 μg) resolved by SDS-PAGE (12.5%) and stained with Coomassie Brilliant Blue.

Experiments in yeast genetics have suggested that eRFs are involved in the translation termination of 2A (19). To examine whether eRFs were required for 2A-2B processing in a mammalian system, we established a protein synthesis system reconstituted with purified eukaryotic factors. To simplify the system, we used the IRES of HCV to promote translation initiation without eIFs; a previous study had shown that the 80 S ribosome bound to the HCV IRES without eIFs at a relatively high concentration of magnesium (13). We expressed recombinant human eEF1s (eEF1A, eEF1Bγ, and eEF1Bα) and ARSs in BHK-21 cells, and we expressed eRF1 and eRF3 in bacteria. These components were then purified (Fig. 4). Endogenous eEF2, the 40 and 60 S ribosomal subunits, and total tRNAs were purified from HeLa cells (Fig. 4). These components were combined in a test tube with amino acids, triphosphate nucleotides (ATP, GTP, CTP, and UTP), and T7 RNA polymerase. The concentration of magnesium was set to 6.0 mm, but the concentration of free magnesium in the system was unknown because the triphosphate nucleotides chelate magnesium (the total nucleotide concentration was 3.7 mm: 1.25 mm ATP and 0.83 mm each of GTP, CTP, and UTP).

**FIGURE 5.**
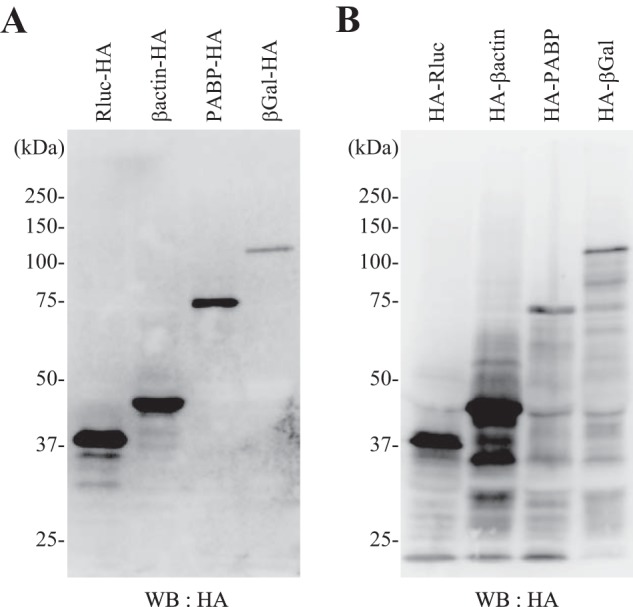
**Expression of various proteins with the translation system reconstituted with human factors.** The components purified in Fig. 4 were combined with T7 RNA polymerase and programmed with a plasmid encoding Rluc-HA, β-actin-HA, PABP-HA, or β-gal-HA (*A*) or encoding HA-Rluc, HA-β-actin, HA-PABP, or HA-β-gal (*B*). Western blotting (*WB*) was performed with the anti-HA antibody after SDS-PAGE (10%).

To examine the efficiency of this reconstituted system, the system was programmed with a plasmid with an ORF for Rluc, β-actin, PABP, or β-gal placed between the HCV IRES and T7 RNA polymerase terminator sequences in the pUC-T7-HCV IRES vector (20). To detect the full-length product, each protein sequence was tagged with HA at the C terminus. After incubation at 32 °C for 3 h, samples were resolved by SDS-PAGE followed by Western blotting with anti HA antibody. This reconstitution system was able to produce the full-length forms (37–120 kDa) of each encoded protein but tended to produce lower amounts of longer polypeptides (Fig. 5*A*). When the HA tag was moved to the N terminus of the same ORFs, several shorter products were detected (Fig. 5*B*). These products were presumably due to premature termination of translation, thereby reducing the yield of the full-length products. Note that because of a high sensitivity of the anti-HA antibody for probing Western blots, only HA-dependent Western blotting was used to monitor protein synthesis with the reconstitution system.

**FIGURE 6.**
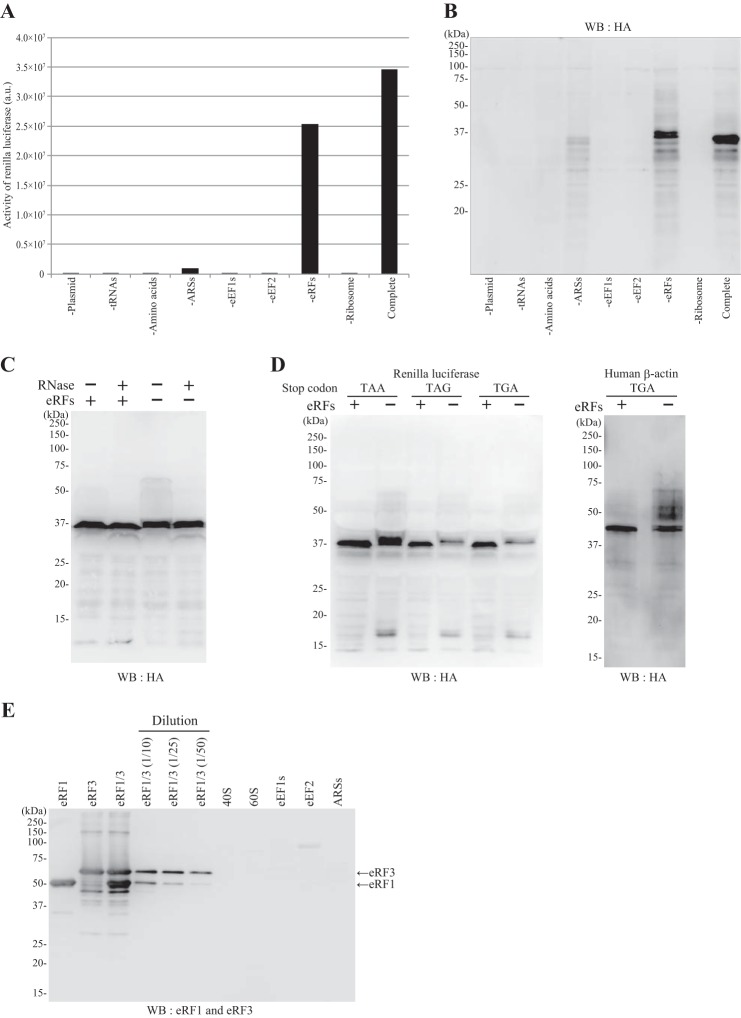
**Dependence of the reconstitution system on each component.** The protein expression system was reconstituted with the components shown in Fig. 4 (*Complete*) or with all but the component listed. The systems were programmed with pUC-T7-HCV-HA-Rluc. After incubation, samples were subjected to the Rluc assay (*A*) and SDS-PAGE (12.5%) followed by Western blotting (*WB*) and probing with the anti-HA antibody (*B*). *C*, effect of RNase treatment. The system was reconstituted with eRFs (+) or without eRFs (−) and programmed with pUC-T7-HCV-HA-Rluc. After translation, each sample was treated with RNase A (final concentration, 0.1 μg/μl) (+) or water (−) for 15 min at 32 °C and resolved by SDS-PAGE (12.5%) followed by Western blotting with anti-HA antibody. *D*, the system was reconstituted with eRFs (+) or without eRFs (−) and programmed with pUC-T7-HCV-HA-Rluc with a termination codon of TAA, TAG, or TGA or programmed with pUC-T7-HCV-HA-β-actin (the termination codon of the β-actin ORF was TGA). *E*, the preparations of the components used in the reconstitution system were analyzed by Western blotting with anti-eRF1 (Cell Signaling Technology) and anti-eRF3 (Sigma) antibodies after SDS-PAGE. The amounts of each component loaded on the gel were the same as those included in one translation reaction (10 μl): eRF1 (0.25 μg), eRF3 (0.25 μg), 40 S subunit (1 μl; 80 *A* units), 60 S subunit (1 μl; 160 *A* units), eEF1s (50 μg), eEF2 (1 μg), and ARSs (3 μg). The sample *eRF1/3* represents a mixture of eRF1 (0.25 μg) and eRF3 (0.25 μg), and the samples diluted 10× (*1/10*), 25× (*1/25*), and 50× (*1/50*) were also examined. *a.u.*, arbitrary units.

To examine the necessity of each ingredient, we withdrew a component from the complete system and programmed it with the HA-Rluc construct. After incubation, aliquots were subjected to the Rluc assay (Fig. 6*A*) and Western blotting (Fig. 6*B*). Withdrawal of ribosomes, tRNAs, eEF1s, or eEF2 resulted in essentially no translation. When ARSs were not included, Rluc was appreciably synthesized albeit weakly because the purified tRNAs most likely included some aminoacylated tRNAs. When eRFs (eRF1 and eRF3) were omitted, only a slight reduction in Rluc activity was observed (Fig. 6*A*), and we detected some up-shifted HA-Rluc products along with the protein of the original mobility on SDS-PAGE (Fig. 6*B*). These up-shifted products were not sensitive to RNase (Fig. 6*C*), suggesting that they were mostly C-terminally elongated products. This C-terminal elongation of the product did not depend on the nucleotide sequence of the termination codon because when we changed the Rluc ORF termination codon (TAA) to TAG or TGA we observed similar C-terminally elongated products (Fig. 6*D*). HA-β-actin (the termination codon of the ORF was TGA) was also C-terminally elongated in the absence of eRFs (Fig. 6*D*). To explain this elongation, we hypothesized that, in the absence of eRFs, a non-cognate aa-tRNA might be aberrantly incorporated into the vacant A site, and translation would continue to the next termination codon or the 3′-end of the mRNA, generating C-terminally elongated products. Indeed, the plasmid construct carries an in-frame termination codon (Table 1). To confirm the dependence of this system on the added eRFs, we examined whether other components in the system were contaminated with eRFs. Western blotting analysis showed that no components purified from cells (ribosomes, ARSs, eEF1, and eEF2) contained detectable levels of eRF1 and eRF3 (Fig. 6*E*). Collectively, the reconstituted translation system that we established depended on all the ingredients for authentic protein synthesis.

**TABLE 1:** **Nucleotide sequences around the C termini of ORFs: HA-Rluc, HA-β-actin, HA-2A-2B-HA, and HA-2A-HA-2B (22 aa)** *, the termination codon of the ORF; **, the T7 terminator: CTAGCATAACCCCTTGGGGCCTCTAAACGGGTCTTGAGGGGTTTTT where the termination codon in-frame to the ORF is underlined; ***, the sequence coding for NPGP is underlined.

ORF	Nucleotide sequence
HA-Rluc	---GATAAATAA*CTCGAGTCTAGA-T7ter**
HA-Rluc (TGA)	---GATAAATGA*CTCGAGTCTAGA-T7ter**
HA-Rluc (TAG)	---GATAAATAG*CTCGAGTCTAGA-T7ter**
HA-β-actin	---TGCTTCTAG*CTCGAGTCTAGA-T7ter**
HA-2A-2B-HA	---GCGGGCTAA*CTCGAGTCTAGA-T7ter**
HA-2A-HA-2B (22 aa)	---AATCCAGGTCCC***TTCCTCGAGTCTAGA-T7ter**

##### 2A-2B Processing Occurs without eRFs

**FIGURE 7.**
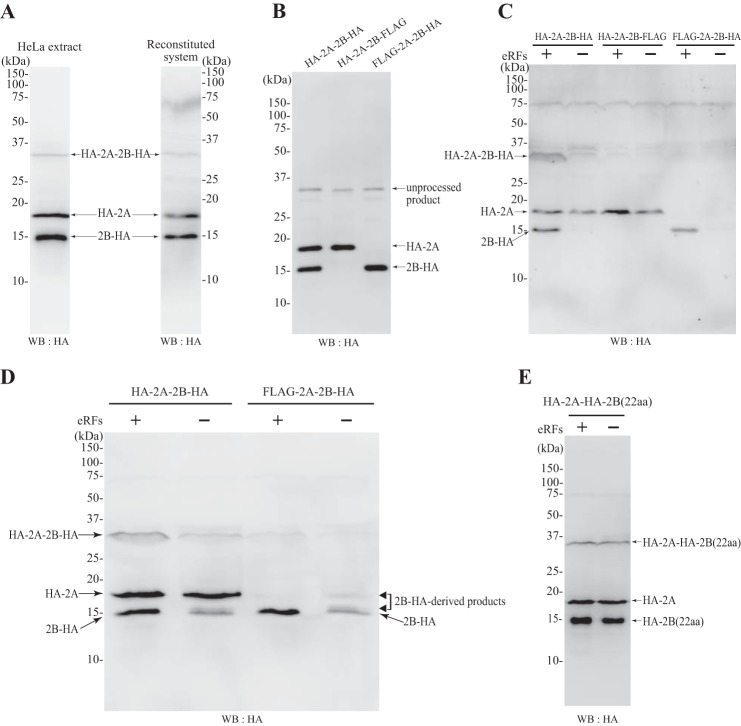
**2A-2B processing does not require eRFs.** *A*, the plasmid pUC-T7-HCV-HA-2A-2B-HA was incubated in a HeLa cell extract-derived protein synthesis system (*left panel*) or in our reconstituted protein expression system with eRFs (*right panel*). *B*, the HeLa cell extract-derived protein expression system was incubated with a template encoding HA-2A-2B-HA, HA-2A-2B-FLAG, or FLAG-2A-2B-HA. *C*, the reconstituted protein expression system was incubated with a template encoding HA-2A-2B-HA, HA-2A-2B-FLAG, or FLAG-2A-2B-HA in the presence (+) or absence (−) of eRFs. *D*, the reconstituted protein expression system was incubated with a template encoding HA-2A-2B-HA or FLAG-2A-2B-HA in the presence (+) or absence (−) of eRFs as in *C*, but the expression system was scaled up 2-fold. *E*, the reconstituted protein expression system was incubated with a template encoding HA-2A-HA-2B (22 aa) in the presence (+) or absence (−) of eRFs. In all cases, Western blotting (*WB*) was performed with anti-HA antibody after SDS-PAGE (12.5%).

After establishing the protein synthesis system reconstituted with human factors, we programmed this system with a template that encoded HA-2A-2B-HA. Western blots probed with anti-HA antibody showed that, like the HeLa cell extract, the reconstituted system was able to process the 2A-2B peptides (Fig. 7*A*); the upper and lower bands correspond to 2A-HA and 2B-HA peptides, respectively (Fig. 7*B*). To examine whether eRFs were required for the processing, eRFs were removed from the reconstituted system and programmed with plasmids encoding HA-2A-2B-HA, HA-2A-2B-FLAG, and FLAG-2A-2B-HA. Western blots were probed with anti-HA antibody (Fig. 7*C*). Synthesis of 2A remained unchanged upon withdrawal of eRFs, but the 2B-HA band was up-shifted, and the intensity of the 2B-HA band of the original mobility was decreased. The 2B-HA-related products were hardly seen on the blot of Fig. 7*C*, but they were clearly detectable in the experiments that were scaled up (Fig. 7*D*). These results suggested that translation of 2A terminated, even when eRFs were omitted, despite the absence of a termination codon; however, translation of 2B-HA terminated at the authentic termination codon, and therefore its translation termination required eRFs. Hence, we constructed a template, HA-2A-HA-2B (22 aa), in which the C-terminal 22 amino acids of 2B were replaced with the C-terminal 20 amino acids of 2A (AHYAG----TNPG) and the N-terminal two amino acids of 2B (PF); this construct was designed to allow the translation of 2B to terminate in the same manner as 2A. Then, to detect the 2B product, we added an HA tag to the N terminus of 2B. When programmed with this template, 2B and 2A were both generated in the presence or absence of eRFs (Fig. 7*E*). These results indicate that 2A-2B processing requires neither eIFs nor eRFs and occurs during the elongation process carried out by the ribosomes, eEF1s, and eEF2.

## DISCUSSION

In this study, we successfully established a protein expression system with purified human factors, and we showed that processing of the EMCV 2A-2B required neither eIFs nor eRFs. Because no cytoplasmic protease was involved, it follows that this processing must occur co-translationally in the ribosome at the elongation step. As proposed previously (18, 36), it is likely that the ribosome terminates translation of 2A and reinitiates translation of 2B without translation initiation or termination codons. Although Doronina *et al.* (19) suggested that eRFs were involved in the processing of FMDV 2A-2B in yeast genetics, no involvement of eRFs as demonstrated in the present study should be reasonable due to the absence of a translation termination codon at the 3′-end of the 2A-coding region. Reinitiation of translation following eRF1/eRF3-dependent termination has been well documented in a toeprinting analysis with a mammalian reconstitution system (37). In that case, the 80 S ribosome with the deacylated tRNA in the P site and no tRNA in the A site migrated bidirectionally to the codon cognate to the P site tRNA. However, it is clear that 2A-2B processing cannot be explained by this mechanism.

Several studies have shown that a nascent peptide chain in the ribosome affects the activity of the ribosome (38): for example, SecM-mediated ribosomal stall (31), the translational pausing of XBP1u mRNA (32), the AdoMet-mediated ribosomal arrest on cystathionine γ-synthase mRNA (39), and the stalling of the ribosome due to the fungal arginine attenuator peptide (40) or the human cytomegalovirus gp48 upstream open reading frame (41). In all of those cases, the ribosome stalled with a peptidyl-tRNA in the P site, allowing detection of the peptidyl-tRNA by gel electrophoresis. In contrast, in the present study, we did not appreciably detect 2A protein ligated with tRNA (Fig. 3). A previous study showed that the ribosome transiently stalled with the FMDV 2A-tRNA^Gly^ in the P site; in that study, after translation in the presence of a low dose of puromycin, an FMDV-2A protein conjugated with puromycin could be isolated with an anti-puromycin antibody (18). Moreover, a toeprint analysis detected a weak signal due to the pausing of the ribosome at the end of the 2A-coding mRNA (19). A possible mechanism is that the C-terminal 2A peptide sequence interacts with the wall of the ribosomal exit tunnel, which affects the PTC activity, and hydrolysis of the 2A tRNA^Gly^ occurs before peptide bond formation. It should be noted that peptide bond formation by the proline residue of prolyl-tRNA^Pro^ in the A site is slower than bonding rates of other amino acids (42). In canonical translation termination, eRF1 recognizes a termination codon in the A site and activates the PTC via the conserved GGQ motif, which then promotes the hydrolysis of the peptidyl-tRNA, and the peptide is released (43). It remains to be determined how the PTC is activated without eRFs when the C-terminal 18 amino acids of 2A are in the ribosomal exit tunnel. Processing of 2A-2B occurred only in the eukaryotic but not prokaryotic systems (Fig. 1) (30), and a eukaryotic-specific loop of the ribosomal protein L10e establishes direct contact with the CCA end of the peptidyl-tRNA (44, 45). Thus, the C-terminal 18 amino acids of 2A might contact L10e, thereby affecting the PTC activity. Nonetheless, we cannot rigorously rule out the possibility that the 2A peptide self-hydrolyzes within the ribosome. Otherwise, a ribosome-associated protein that could not be removed during purification of ribosomes might be involved in 2A-2B processing.

The reconstituted translation system established in this study was capable of synthesizing proteins as large as 120 kDa. The initiation phase was simplified by introducing the HCV IRES (13), thereby circumventing purification of numerous eIFs. In this regard, addition of ABCE1 and Ligatin, which were shown to be responsible for ribosome recycling in eukaryotic translation (46, 47), did not enhance the efficiency of protein synthesis in our system (data not shown). This result was probably due to the lack of eIFs in the system; ribosome recycling occurs as a collaboration between termination and initiation of translation (12). Finally, although this system may not be useful for studying translation initiation, it enables investigation of the elongation and termination phases of translation by the eukaryotic ribosomal complex and of post-translational events like co-translational protein folding.

## Footnotes

3 The abbreviations used are:

PURE: protein synthesis using recombinant elements
EMCV: encephalomyocarditis virus
HCV: hepatitis C virus
IRES: internal ribosome entry site
eRF: eukaryotic release factor
eEF: eukaryotic elongation factor
eIF: eukaryotic translation initiation factor
FMDV: foot-and-mouth disease virus
PTC: peptidyltransferase center
Rluc: *Renilla* luciferase
PABP: poly(A)-binding protein
aa: amino acid(s)
uORF2: upstream open reading frame 2
ARS: aminoacyl-tRNA synthetase.
